# Supplementary material for: A large-scale trial in Chennai, India shows effective reduction of Anopheles stephensi populations by the biolarvicide Natular
Source: PLoS One. 2025 Dec 15;20(12):e0337845. doi: 10.1371/journal.pone.0337845 (PMC12704891; doi:10.1371/journal.pone.0337845)
Supplement: Attachment — Submitted filename: 03. Response to reviewers_PONE-D-24-057840 05.11.2025.docx [file pone.0337845.s004.docx]

| **Sl. No.** | **Comments** | **Clarifications/Answers** |
| --- | --- | --- |
| 1 | Please ensure that your manuscript meets PLOS ONE's style requirements, including those for file naming. The PLOS ONE style templates can be found at  <https://journals.plos.org/plosone/s/file?id=wjVg/PLOSOne_formatting_sample_main_body.pdf> and  <https://journals.plos.org/plosone/s/file?id=ba62/PLOSOne_formatting_sample_title_authors_affiliations.pdf> | The manuscript has been revised in accordance with the PLoS ONE formatting and style requirements. |
| 2 | In your Methods section, please provide additional information regarding the permits you obtained for the work. Please ensure you have included the full name of the authority that approved the field site access and, if no permits were required, a brief statement explaining why. | As per the reviewer’s suggestion, the details regarding the permission have been incorporated into the Methodology section. (Page 6; Lines 108-111), |
| 3 | In the online submission form, you indicated that "The data is available in the manuscript. However, the data would be made available upon request."  All PLOS journals now require all data underlying the findings described in their manuscript to be freely available to other researchers, either 1. In a public repository, 2. Within the manuscript itself, or 3. Uploaded as supplementary information.  This policy applies to all data except where public deposition would breach compliance with the protocol approved by your research ethics board. If your data cannot be made publicly available for ethical or legal reasons (e.g., public availability would compromise patient privacy), please explain your reasons on resubmission and your exemption request will be escalated for approval. | All the data is available in the manuscript itself. |
| 4 | We note that Figure 1 in your submission contain map/satellite images which may be copyrighted. All PLOS content is published under the Creative Commons Attribution License (CC BY 4.0), which means that the manuscript, images, and Supporting Information files will be freely available online, and any third party is permitted to access, download, copy, distribute, and use these materials in any way, even commercially, with proper attribution. For these reasons, we cannot publish previously copyrighted maps or satellite images created using proprietary data, such as Google software (Google Maps, Street View, and Earth). For more information, see our copyright guidelines: <http://journals.plos.org/plosone/s/licenses-and-copyright>. We require you to either (a) present written permission from the copyright holder to publish these figures specifically under the CC BY 4.0 license, or (b) remove the figures from your submission | As suggested, the replacement figure has been prepared and incorporated into the revised manuscript. |
| 5 | Please review your reference list to ensure that it is complete and correct. If you have cited papers that have been retracted, please include the rationale for doing so in the manuscript text, or remove these references and replace them with relevant current references. Any changes to the reference list should be mentioned in the rebuttal letter that accompanies your revised manuscript. If you need to cite a retracted article, indicate the article’s retracted status in the References list and also include a citation and full reference for the retraction notice. | As per the reviewer’s suggestion, the references have been thoroughly reviewed and corrected (Page 22-26). |
| **Additional Editor Comments:** | | |
| 1 | Authors need to address orphan claims in the discussion sections with fig number or cited reference. The stats applied needs to be explained in terms of significance. Optionally, a more mechanistic insight into the observed phenomenon seems to be lacking in the discussion section. The manuscript needs revision according to the expert reviewers. There are no suggested further/revised experiments, and therefore a minor revision is recommended. | We thank the reviewer for highlighting this important aspect. As suggested, the relevant citations have been added (Page 17 & 18; Lines 415 & 418), and the corresponding figure numbers have been provided (Page 18-20; Lines 424-225, 431, 442-443, 453-455, 464-465, 471). In addition, an explanation of the statistical analyses has been included in the Discussion section (Page 18-20; Lines 422-486). We believe these additions have enhanced the clarity and value of the Discussion in the revised manuscript. |
| **Review Comments to the Author** | | |
| 1 | **Reviewer #1:** The manuscript needs to be revised to improve it. The title needs to be more concise, the abstract needs to correctly capture the findings and statements of statistical significance of means of populations will be important. There are also certain questions that have to be addressed probably in the discussion section. A more detailed file with comments to the authors is attached. | We thank the reviewer for their valuable comments. As suggested, the manuscript has been revised accordingly. A concise title has been provided (Page 1) and the abstract has been modified (Page 2; Lines 1-20), and an explanation of the statistical analysis has been included in the Discussion section (Page 18-20; Lines 422-486). We believe that incorporating these suggestions has further improved the overall quality of the manuscript. |
| 2 | **Reviewer #2:**  The authors don’t discuss the mechanism of action for the three different formulations.  Have any cross-resistance studies been conducted for the formulations. | The mechanism of action of Spinosad involves disrupting the function of ion channels regulated by GABA and nicotinic adenosine triphosphate (nAChR), leading to depolarization and subsequent excitation of insect neurons (included in the Methods – Test Materials section Page 5; Lines 89-96).  Although the mechanism of action of Spinosad remains the same across different formulations of Natular, the rate and pattern of active ingredient release into breeding habitats vary depending on the formulation type. To date, no cross-resistance studies have been conducted on these formulations against *Anopheles stephensi*. |
| **Reviewers' comments:** | | |
| 1. | **Title page**  A large-scale trial in Chennai, India found that Natular, a biolarvicide, was effective in reducing populations of *Anopheles stephensi*, a major malaria vector  **Comment:** Change the title to “A large-scale trial in Chennai, India shows effective reduction of *Anopheles stephensi* populations by the biolarvicide Natular” | As per the reviewer’s suggestion, the title has been appropriately revised (Page 1). |
| 2 | **Study area:**  Tamil Nadu, India, under the jurisdiction of the Greater Chennai Corporation (GCC). Chennai is geographically located at 13° 5' 24" N and 80° 16' 12" E along the Coromandel Coast, in the northern region of Tamil Nadu (citation?).  Presently, the estimated population of the city is 7.1 million, distributed among three regions: North Chennai, Central Chennai, and South Chennai. The city  encompasses a total area of 426 square kilometres (citation?). | As suggested, the references have been incorporated in the revised manuscript (Page 6; Lines 103 & 105). |
| 3 | Due to its littoral and equatorial location, the city experiences minimal fluctuations in seasonal temperatures. Minimum temperatures in January range between 18 and 20 degrees Celsius, whereas maximum  temperatures in May and June reach 38 to 42 degrees Celsius (citation?).  The northeast monsoon, which  occurs between mid-October and mid-December, provides the majority of the annual precipitation of about 1400 millimetres (citation?).  Annually, between 53.6% and 78.8% of the state's malaria cases have been reported  in the city alone over the last two decades (citation?).  As of October 2017, 3,303 of the total 4,671 malaria  cases in the state were reported in Chennai. Chennai documented a total of 1,471 malaria cases  in 2018 and 771 malaria cases in 2019, with Plasmodium vivax constituting the prevailing  parasite species, accounting for 95 to 97% of the reported cases (citation?). | As suggested, the references have been incorporated in the revised manuscript (Page 6; Lines 118, 120, 124 & 128). |
| 4 | In 500-liter overhead (sintex) tanks (OHTs), Natular formulations were evaluated against *An. stephensi* at the optimum field application dosage determined in the phase II trial (citation?). | As suggested, the reference has been included (ICMR-VCRC, unpublished data) (Page 7; Line 145). |
| 5 | **Comment:** what was the value of the optimum field application dosage determined in the phase II trial and could you give a citation? | The optimal field application dosages for the selected habitats are as follows: 1) 0.274 mg active ingredient (AI)/L for the 20.6% EC formulation, 2) 0.06 mg AI/L for the 2.5% G30 formulation, and 3) 1 tablet per 200 L for the 7.48% DT formulation. The results of the Phase II trial have not yet been published; therefore, they are cited as VCRC, unpublished report. As suggested, the optimum field application dosages determined during the Phase II trial have been included for the various formulations (ICMR-VCRC, unpublished data). |
| 6 | **Results**  **General comment:**  The values for reduction of mosquito population by spinosad in the abstract were not consistent with those presented in the results section. The authors should modify the abstract accordingly. | As per the reviewer’s suggestion, the values reflecting the reduction in mosquito populations by Spinosad have been appropriately revised in the Abstract section (Page 2) |
| 7 | **Results**  **General comment:**  In the text, include statements of the statistical significance of the 3 different test formulations and their respective control populations for the seasons investigated. Give the p values as appropriate. | Agreed. We have provided an explanation of the statistical analyses, which has been included in the Discussion section (Page 18-20; Lines 422-486). |
| 8 | **Discussion**  In a recent publication, Clarke Environmental Technologies (I) Ltd., located in Mumbai, India, introduced three novel spinosad (Natular) formulations:  7.48% DT, 20.6% EC, and 2.5% G30 (citation?). These formulations demonstrated efficacy against *An. stephensi* larvae in both simulated field (phase II) and laboratory (phase I) conditions (ICMR VCRC, unpublished report).  **Comment:** Could the authors cite the recent publication from Clarke Environmental Technologies? | As per the reviewer’s suggestion, references to earlier studies on the evaluation of different Natular formulations have been added (Page 18; Line 418). |
| 9 | **Conclusions:**  Furthermore, in line with the Integrated Vector Management program, natular (spinosad) formulations appear to be effective biolarvicides for managing *An. stephensi* and other container-breeding mosquitoes, as evidenced by their longer residual activity, efficacy, and favorable toxicological and environmental profile.  **Comment:** Limit conclusion to *An. stephensi* since other container-breeding mosquitoes were not investigated in this study. | As per the reviewer’s suggestion, the conclusions have been appropriately revised (Page 21; Line 502-503). |
| 10 | **References**  **Comment:** Check to make sure that all references comply with the journal referencing style. | As suggested, all the references have been revised to conform to the journal’s referencing style (Page 22-26). |
| 11 | **General questions:** Given that spinosad has been in existing use for several years, what is the significance of this particular study?  How will the results from this study translate into real life field applications?  While the 3 formulations show varying efficacy in different seasons, the authors do need to recommend to readers which of the 3 formulations is the best and why. Will these 3 formulations be purchased or given free to residents to put in their water tanks, wells and sumps around their surroundings, and what are short or long term health risks of spinosads use in domestic water to humans? | Spinosad has been in use since 1999 for agricultural pest control on more than 250 crops and for consumer and animal health applications in over 85 countries. Its introduction into public health began in 2009, when it was developed as the active ingredient in Clarke’s Natular® line of mosquito larvicides.    The novelty and significance of the present study lie in evaluating the efficacy of multiple Natular® formulations of Spinosad (DT, GR, and EC) against *Anopheles* *stephensi* under field conditions in India—an aspect that has not been previously explored. Although Spinosad’s mode of action is well known, its comparative performance and persistence across different formulations and seasons in An. *stephensi* breeding habitats were not documented earlier. The findings from this study therefore provide evidence-based guidance for selecting the most suitable formulation for operational vector control programs in urban malaria settings.  Among the three tested formulations, Natular® 7.48% DT demonstrated the best performance, maintaining larvicidal efficacy for up to 8 weeks at a dosage of one tablet per 200 L of water, making it more practical and cost-effective for field application than the granular or emulsifiable concentrate (EC) formulations. Its extended residual activity reduces the frequency of application, thereby enhancing the feasibility of larviciding within integrated vector management (IVM) programs.  In terms of real-world application, Natular® DT can be operationally deployed by vector control agencies in overhead tanks, wells, sumps, and other domestic water storage containers where *An. stephensi* commonly breeds. It is unlikely to be distributed directly to residents for personal use but rather applied through supervised public health programs following WHO and national guidelines.  Regarding safety, Spinosad was the first mosquito larvicide active ingredient registered under the U.S. EPA’s Reduced Risk Program due to its low toxicity to humans, animals, and non-target organisms. It is non-carcinogenic, non-genotoxic, and not a reproductive or developmental toxin. When used as per the manufacturer’s label instructions, Natular® products pose minimal risk to human health or the environment. The EPA has approved Spinosad formulations for use in a range of aquatic habitats, including residential and recreational areas (according to the manufacturer, Clarke International, USA).  In summary, this study provides novel, field-based evidence supporting the suitability of Natular® 7.48% DT for sustained *An. stephensi* control in Indian urban settings, with demonstrated safety and operational feasibility for inclusion in vector control programs. |
