# Supplementary material for: A large-scale trial in Chennai, India shows effective reduction of Anopheles stephensi populations by the biolarvicide Natular
Source: PLoS One. 2025 Dec 15;20(12):e0337845. doi: 10.1371/journal.pone.0337845 (PMC12704891; doi:10.1371/journal.pone.0337845)
Supplement: Attachment — Submitted filename: PONE-D-24-05784 reviewer comments.docx [file pone.0337845.s001.docx]

PONE-D-24-05784 reviewer comments

**Title page**

A large-scale trial in Chennai, India found that Natular, a biolarvicide, was effective in reducing populations of *Anopheles stephensi*, a major malaria vector

**Comment**: Change the title to “A large-scale trial in Chennai, India shows effective reduction of *Anopheles stephensi* populations by the biolarvicide Natular”

**Abstract**

4 use in mosquito control remain limited. This study aimed to assess the effectiveness of three

5 ~~newly formulated~~ formulations of the biolarvicide~~s,~~ Natular, namely the 20.6% Emulsifiable Concentrate (EC), 2.5% G30

6 granular, and 7.48% tablet for direct application (DT), against *Anopheles stephensi*

7 ~~Liston,1901,~~ in large-scale field trials conducted in Chennai, India.

9 breeding sites, it led to a ~~remarkable~~ >90-100% reduction in *An. stephensi* larval and pupal

**Introduction**

55 Clarke Environmental Technologies (I) Ltd., a company based in Mumbai, India, has released

56 three new formulations of Natular to go along with these changes: Extended Release Bi-Layer

57 Tablet (7.48% DT), Emulsifiable Concentrate (20.6% EC), and Extended Release Granule

58 (2.5% DT). The formulations of Natular contain spinosad as their active ingredient and ~~These formulations~~ are developed for evaluation against mosquito vectors in

59 diverse ecological settings.

60 ~~The formulations of Natular contain spinosad as their active ingredient.~~ In Puducherry, India,

61 the Indian Council of Medical Research-Vector Control Research Centre (ICMR-VCRC)

62 assessed the efficacy of these formulations against Anopheles stephensi Liston immature stages

63 in Phase I (laboratory) and Phase II (small-scale field) evaluations. The objective of the present

**Materials & methods**

77 In India, three formulations of Natular (7.48% DT, 20.6% EC, and 2.5% G30) were supplied

78 by the manufacturer, Clarke Environmental Technologies (I) Ltd., ~~situated~~ located in Mumbai, India,

79 for the large-scale (Phase III) evaluation against *An. stephensi*.

82 Tamil Nadu, India, under the jurisdiction of the Greater Chennai Corporation (GCC). Chennai

83 is geographically located at 13° 5' 24" N and 80° 16' 12" E along the Coromandel Coast, in the

84 northern region of Tamil Nadu (citation?). Presently, the estimated population of the city is 7.1 million,

85 distributed among three regions: North Chennai, Central Chennai, and South Chennai. The city

86 encompasses a total area of 426 square kilometres (citation?).

91 High temperatures and high levels of humidity are distinctive features of Chennai's tropical wet

92 and dry climate ~~(Koppen: Aw)~~ for the majority of the year. Due to its littoral and equatorial

93 location, the city experiences minimal fluctuations in seasonal temperatures. Minimum

94 temperatures in January range between 18 and 20 degrees Celsius, whereas maximum

95 temperatures in May and June reach 38 to 42 degrees Celsius (citation?). The northeast monsoon, which

96 occurs between mid-October and mid-December, provides the majority of the annual

97 precipitation of about 1400 millimeters (citation?).

100 to October. Annually, between 53.6% and 78.8% of the state's malaria cases have been reported

101 in the city alone over the last two decades (citation?). As of October 2017, 3,303 of the total 4,671 malaria

102 cases in the state were reported in Chennai. Chennai documented 102 a total of 1,471 malaria cases

103 in 2018 and 771 malaria cases in 2019, with Plasmodium vivax constituting the prevailing

104 parasite species, accounting for 95 to 97% of the reported cases (citation?).

119 In 500-liter overhead (sintex) tanks (OHTs), Natular formulations were evaluated against An.

120 stephensi at the optimum field application dosage determined in the phase II trial (citation?).

**Comment:** what was the value of the optimum field application dosage determined in the phase II trial and could you give a citation?

121 evaluation, a total of 122 OHTs breeding with An. stephensi were selected. 76 were randomly

122 assigned to receive treatment with 20.6% EC [28 in winter (W), 24 in rainy weather (R), and

123 24 in summer (S)]. The remaining 46 ~~individuals~~ (16 in R, 15 in W, and 15 in S) served as

**Results**

**General comment:**

1. The values for reduction of mosquito population by spinosad in the abstract were not consistent with those presented in the results section. The authors should modify the abstract accordingly.

2. In the text, include statements of the statistical significance of the 3 different test formulations and their respective control populations for the seasons investigated. Give the p values as appropriate.

**Discussion**

290 are utilized as larvicides in order to control the larval population. Nevertheless, previous studies

291 have documented temephos resistance [25]. Besides, chemical insecticides present health and environmental risks.

292 ~~When this situation arises~~ To avoid these situations, the biolarvicide spinosad (Natular) ~~is viewed~~ can be used as an appropriate

293 replacement for traditional chemical larvicides. The World Health Organization Pesticide

296 8.33% DT, 0.5% GR, and 12% SC. In a recent publication, Clarke Environmental Technologies

297 (I) Ltd., located in Mumbai, India, introduced three novel spinosad (Natular) formulations:

298 7.48% DT, 20.6% EC, and 2.5% G30 (citation?). These formulations demonstrated efficacy against An.

299 stephensi larvae in both simulated field (phase II) and laboratory (phase I) conditions (ICMR300

VCRC, unpublished report).

**Comment:** Could the authors cite the recent publication from Clarke Environmental Technologies?

**Conclusions**

315 currently in use. Furthermore, in line with the Integrated Vector Management program, natular

316 (spinosad) formulations appear to be effective biolarvicides for managing *An. stephensi* ~~and~~

~~317 other container-breeding mosquitoes~~, as evidenced by their longer residual activity, efficacy,

318 and favorable toxicological and environmental profile.

**Comment:** Limit conclusion to *An. stephensi* since other container-breeding mosquitoes were not investigated in this study.

**References**

**Comment:** Check to make sure that all references comply with the journal referencing style.

**General questions:** Given that spinosad has been in existing use for several years, what is the significance of this particular study? How will the results from this study translate into real life field applications? While the 3 formulations show varying efficacy in different seasons, the authors do need to recommend to readers which of the 3 formulations is the best and why. Will these 3 formulations be purchased or given free to residents to put in their water tanks, wells and sumps around their surroundings, and what are short or long term health risks of spinosads use in domestic water to humans?
